# Macrophages produce and functionally respond to interleukin-34 in colon cancer

**DOI:** 10.1038/s41420-020-00350-7

**Published:** 2020-11-05

**Authors:** Eleonora Franzè, Federica Laudisi, Antonio Di Grazia, Martin Marônek, Vittoria Bellato, Giuseppe Sica, Giovanni Monteleone

**Affiliations:** 1 Department of Systems Medicine, University of Rome “TOR VERGATA”, Rome, Italy; 2 Institute of Molecular Biomedicine, Faculty of Medicine, Comenius University in Bratislava, Bratislava, Slovakia; 3 Department of Surgery, University “TOR VERGATA” of Rome, Rome, Italy

**Keywords:** Cancer microenvironment, Translational research

## Abstract

In colorectal cancer (CRC), macrophages represent a major component of the tumor mass and exert mostly functions promoting tumor cell survival, proliferation, and dissemination. Interleukin-34 (IL-34) is a cytokine overproduced by colon cancer (CRC) cells and supposed to make a valid contribution to the growth and diffusion of CRC cells. The biological functions of IL-34 are mediated by the macrophage colony-stimulating factor receptor (M-CSFR-1), which controls monocyte/macrophage differentiation, growth, and survival. We here investigated whether, in CRC, tumor-associated macrophages (TAMs) express M-CSFR-1 and functionally respond to IL-34. By flow-cytometry analysis of tumor-infiltrating cells (TICs) and lamina propria mononuclear cells (LPMCs) isolated from normal, adjacent mucosa of CRC patients, we showed that CD68/HLA-DRII-expressing TICs and LPMCs expressed M-CSFR-1. Both these cell types produced IL-34 even though the expression of the cytokine was more pronounced in TICs as compared to normal LPMCs. Moreover, in CRC samples, there was a positive correlation between IL-34-producing cells and CD68-positive cells. Stimulation of LPMCs and TICs with IL-34 resulted in enhanced expression of CD163 and CD206, two markers of type II-polarized macrophages, and this was evident at both RNA and protein level. In the same cell cultures, IL-34 stimulated expression and production of IL-6, a cytokine known to promote CRC cell growth and diffusion. Finally, knockdown of IL-34 in TICs with specific antisense oligonucleotides with: a specific antisesne oligonucleotide decreased IL-6 production and the number of TAMs producing this cytokine. This is the first to show a positive role of IL-34 in the control of TAMs in CRC, further supporting the notion that IL-34 sustains colon tumorigenesis.

## Introduction

Colorectal carcinoma (CRC), one of the most common forms of malignancy in the western world, is supposed to be caused by a complex interaction between environmental carcinogens and genetic alterations, which ultimately results in the uncontrolled growth of transformed cells^1^. Approximately 2% of CRC arise in patients with long-standing inflammatory bowel disease (IBD), while most CRC develop in individuals who are not affected by IBD^2^. However, even in these latter patients, the cancer tissue is infiltrated with various immune cells, which can either promote or inhibit CRC cell growth^3^. The promoting effect of immune cells on colon carcinogenesis is largely mediated by cytokines and growth factors, which either directly or indirectly trigger proliferative signals in CRC cells^4^. Within the tumor tissue, other cell types (i.e., stromal cells and cancer cells themselves) can secrete factors promoting CRC cell growth and survival^5^. For instance, we have recently shown that interleukin-34 (IL-34), a cytokine that regulates monocyte/macrophage functions, is highly produced in CRC^6,7^. Immunohistochemistry analysis of CRC sections showed that cancer cells were the major source of IL-34, even though it was also produced to a lesser extent by lamina propria mononuclear cells (LPMCs)^6^. CRC cells expressed both the macrophage colony-stimulating factor receptor (M-CSF1-R) and PTP-z, two functional IL-34 receptors, suggesting that IL-34 can act as an autocrine and/or paracrine factor that targets cancer cells in vivo^6^. Indeed, stimulation of CRC cells with IL-34 enhanced cell growth and invasion through an ERK1/2 MAP kinase-dependent mechanism^6^.

The tumor mass contains many cell types (i.e., neoplastic cells, fibroblasts, endothelial, and immune cells), which interact reciprocally with the downstream effect of sustaining cancer cell growth, survival, and diffusion^8^. Macrophages represent up to 50% of the tumor mass, and some subsets of them promote tumor cell survival, proliferation, and dissemination^9^. Indeed, high numbers of tumor-associated macrophages (TAMs) correlate often with a bad prognosis^10^. TAMs originate from blood monocytes, which are recruited at the tumor tissue, where they differentiate mostly in type 2-polarized (or alternatively activated) macrophages^11^. Factors/mechanisms involved in the differentiation and activation of type 2-polarized TAMs in CRC are not fully understood. We here investigated whether, in CRC, TAMs produce and functionally respond to IL-34.

## Results

### TAMs express IL-34 receptors and produce IL-34

**Fig. 1:**
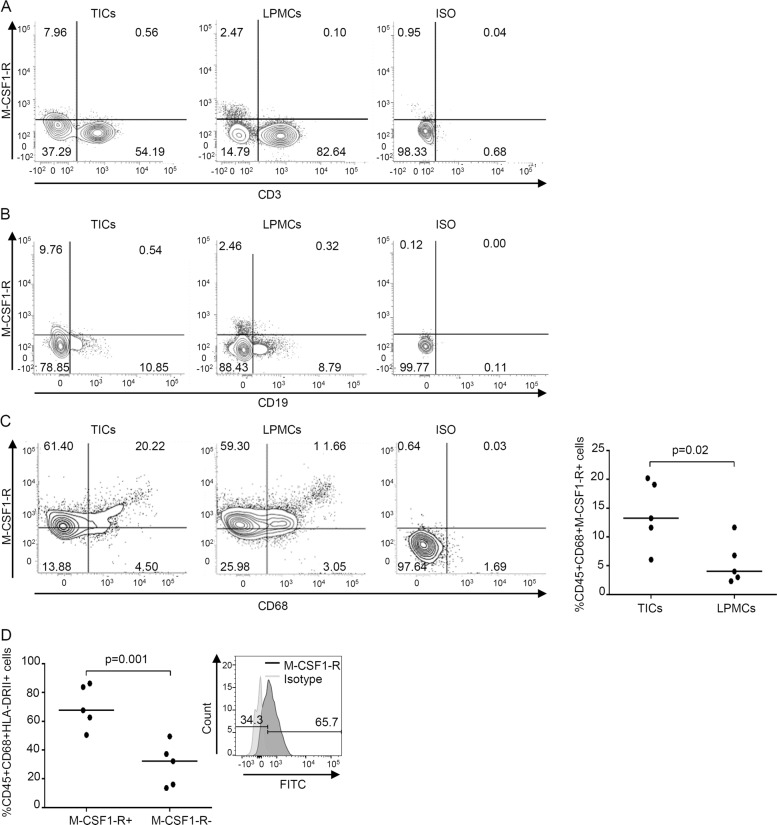
Expression of macrophage colony-stimulating factor 1 receptor (M-CSF1-R) is increased in intestinal CD68 + HLA-DR + tumor-infiltrating cells (TICs). **A**, **B** Representative dotplots showing M-CSF1-R-expressing cells in TICs or lamina propria mononuclear cells (LPMCs) isolated, respectively, from tumoral and non-tumoral areas of surgical samples of one patient with colon rectal cancer (CRC). Cells were gated on live CD45 + cells and subsequently analyzed for the expression of M-CSF1-R and CD3 (**A**) or CD19 (**B**) by flow cytometry. One of five representative experiments is shown. Staining with the respective isotype control antibodies is also shown. **C** Representative dotplots showing M-CSF1-R-expressing cells in TICs or LPMCs isolated as above. Cells were gated on CD45 + live cells and subsequently analyzed for the expression of M-CSF1-R and CD68 by flow cytometry. The right panel shows live CD45 + CD68 + cells expressing M-CSF1-R in TICs or LPMCs isolated from five patients with CRC. Each point in the graph indicates the percentage of live CD45 + CD68 + cells expressing M-CSF1-R in a single sample of a single CRC patient. Horizontal bars indicate the median values. **D** Intestinal TICs were isolated from tumoral areas of five patients with CRC, and M-CSF1-R expression was analyzed in live CD45 + CD68 + HLA-DRII + cells by flow cytometry. Each point in the graph indicates the percentage of live CD45 + CD68 + HLA-DRII + cells either expressing or not M-CSF1-R in a single sample of a single CRC patient. Horizontal bars indicate the median values. Right panel: representative histograms showing the expression of M-CSF1-R in TICs isolated from tumoral area of one patient with CRC and analyzed by flow cytometry. The numbers indicate the percentage of live CD45 + CD68 + HLA-DRII + cells, either positive or negative, for M-CSF1-R. Histograms with isotype control antibodies are also shown.

To begin to examine whether, in CRC, TAMs are a cell target of IL-34, we isolated TICs from CRC tissues and LPMCs from normal, adjacent mucosal areas of CRC patients and examined them by flow cytometry. By gating on live CD45 + cells, we showed that M-CSF1-R was barely detectable in CD3 + cells and CD19 + cells (Fig. 1A, B), while the receptor was expressed by CD68 + cells (Fig. 1C). The percentage of CD45 + CD68 + cells expressing M-CSF1-R was significantly greater in TICs than in LPMCs (Fig. 1C, right inset). When the analysis was restricted to TICs, it was evident that more than 50% of CD68 + HLA-DRII + cells, which correspond to TAMs, expressed the receptor, and the percentage of such cells was significantly greater than the percentage of cells negative for M-CSF1-R (Fig. 1D).

**Fig. 2:**
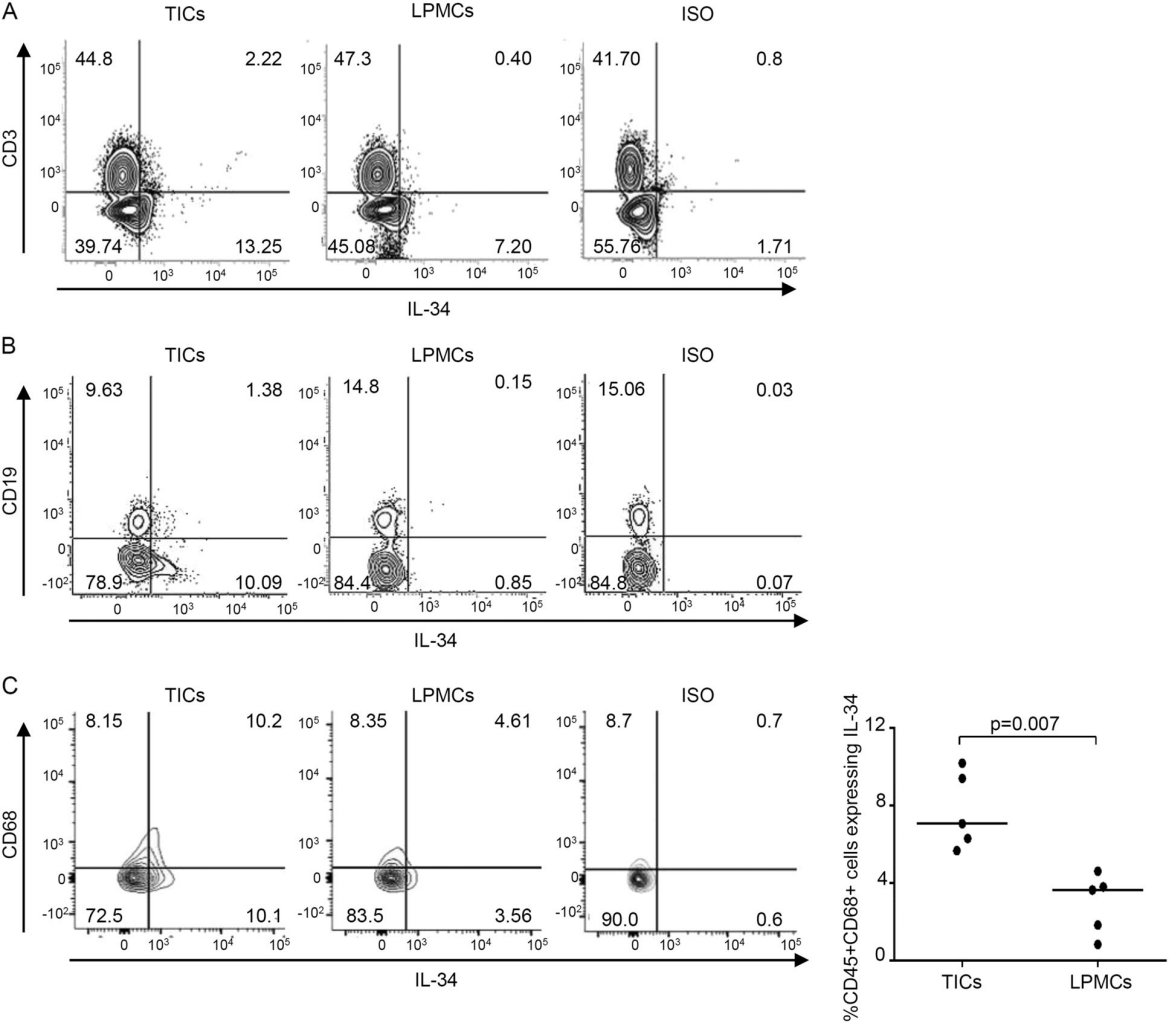
Interleukin-34 (IL-34) is produced by intestinal CD68 + tumor-infiltrating cells (TICs). **A**, **B** Representative dotplots showing IL-34-producing cells in TICs or lamina propria mononuclear cells (LPMCs) isolated, respectively, from the tumoral and non-tumoral areas of one patient with CRC. Cells were gated on live CD45 + cells and subsequently analyzed for the expression of IL-34, CD3 (**A**) or CD19 (**B**) by flow cytometry. One of five representative experiments is shown. Staining with the IL-34 isotype control antibodies is also shown. **C** Representative dotplots showing IL-34-producing CD68 + TICs or LPMCs isolated as above. Cells were gated on CD45 + live cells and subsequently analyzed for the expression of IL-34 and CD68 by flow cytometry. The right panel shows live CD45 + CD68 + cells expressing IL-34 in TICs or LPMCs isolated from five patients with CRC. Each point in the graph indicates the percentage of live CD45 + CD68 + cells expressing IL-34 in a single sample of a single CRC patient. Horizontal bars indicate the median values.

Next, we determined the cell source of IL-34 in CRC tissue. Flow-cytometry analysis of TICs and LPMCs preparations showed that ~2% of CD3 + cells and CD19 + TICs were positive for IL-34, while only a few CD3 + and CD19 + LPMCs expressed IL-34 (Fig. 2A, B). A more prevalent expression of IL-34 was seen in CD68 + cells, and the percentage of CD68/IL-34-expressing cells was significantly greater in TICs than in LPMCs (Fig. 2C).

**Fig. 3:**
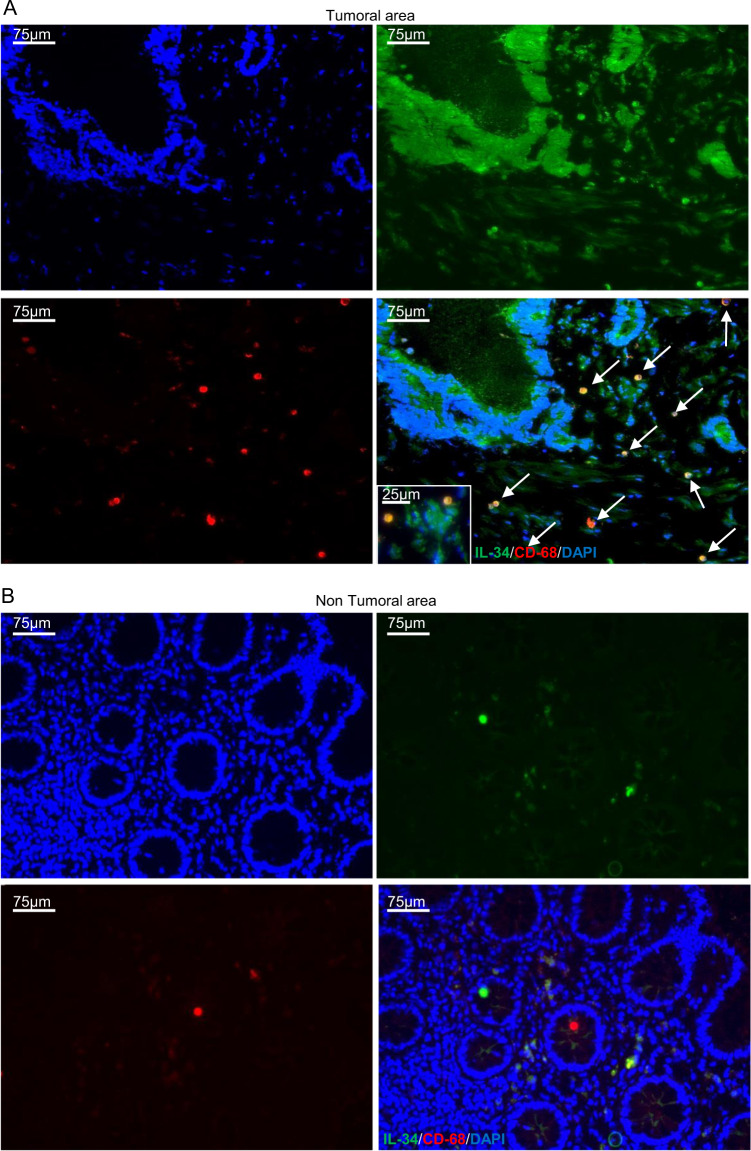
IL-34 is expressed by CD68-positive cells in CRC. Representative images of double-immunofluorescence staining of colon sections taken from tumoral and non-tumoral areas of one patient with CRC and analyzed for the expression of IL-34 (green), CD68 (red), and DAPI (blue). The scale bars are 75 µm. Arrows indicate cells co-expressing IL-34 and CD68. Images at higher magnification (25 um) are also shown.

**Fig. 4:**
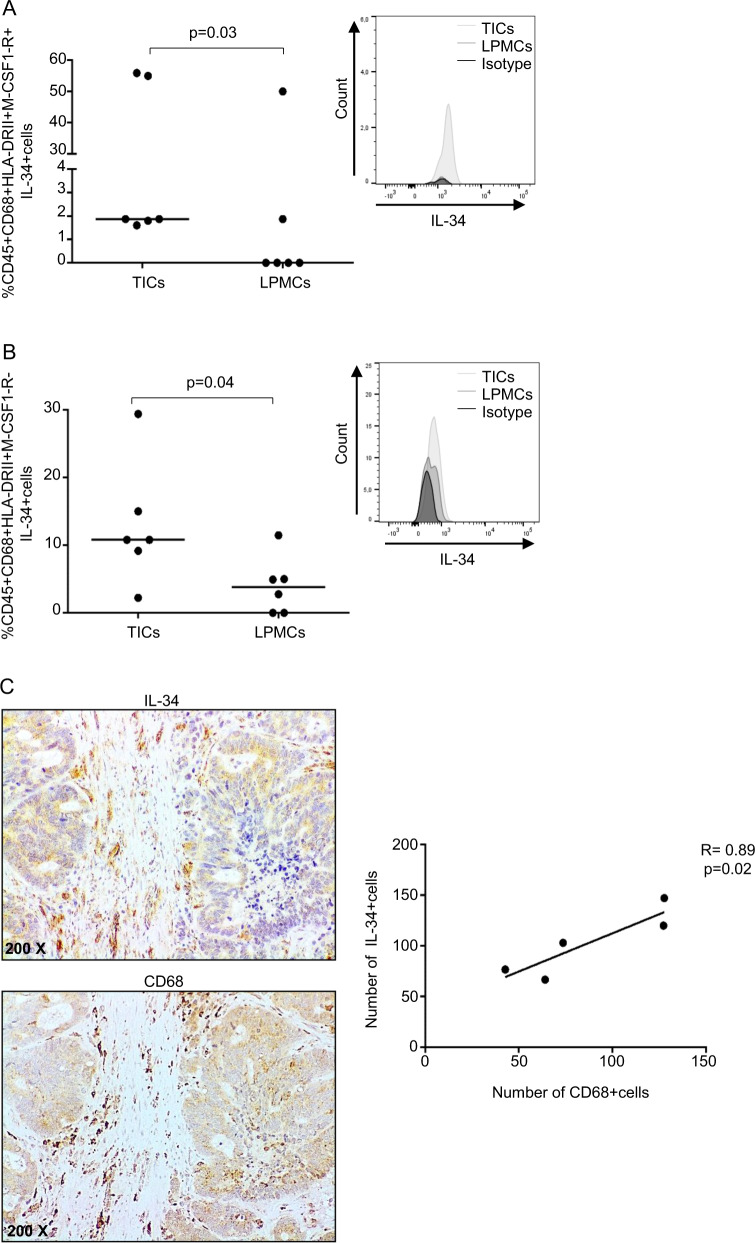
Tumor-associated macrophages (TAMs) produce IL-34. **A**, **B** Intestinal TICs and LPMCs were isolated, respectively, from tumoral and non-tumoral samples of six patients with CRC and analyzed for the percentage of live CD45 + CD68 + HLA-DRII + IL-34+ cells either expressing (**A**) or not (**B**) M-CSF1-R + by flow cytometry. Each point in the graph indicates the percentage of positive cells in a single sample of a single CRC patient; horizontal bars indicate the median values. Right panels: representative histograms showing IL-34 expression in live CD45 + CD68 + HLA-DRII + TICs and LPMCs either expressing (**A**) or not (**B**) M-CSF1-R. Staining with isotype control antibodies is also shown. **C** Representative photomicrographs (×200 original magnification) of serial formalin-fixed, paraffin-embedded colon sections of surgical samples taken from the tumoral area of one patient with CRC and stained with IL-34 or CD68. The right panel shows the correlation between the number of IL-34-expressing cells and the number of CD68-positive cells as evaluated by immunostaining in frozen sections of surgical specimens taken from five CRC patients.

Immunofluorescence analysis confirmed that IL-34 is produced by CD68 + cells in the tumoral area of CRC patients (Fig. 3). Further analysis showed that the percentages of CD68 + HLA-DRII + IL-34+ either expressing or not M-CSF1-R were significantly greater in TICs than in LPMCs (Fig. 4A, B) even though in both cell preparations there was variability in the percentage of IL-34-expressing cells (Fig. 4A, B). Finally, by immunohistochemistry of CRC sections, we showed a positive correlation between the number of CD68 + cells and the number of IL-34-producing cells (Fig. 4C).

Altogether these data indicate that macrophages infiltrating CRC tissue produce IL-34 and express IL-34 receptor.

### IL-34 enhances the expression of markers of type 2-polarized macrophages

**Fig. 5:**
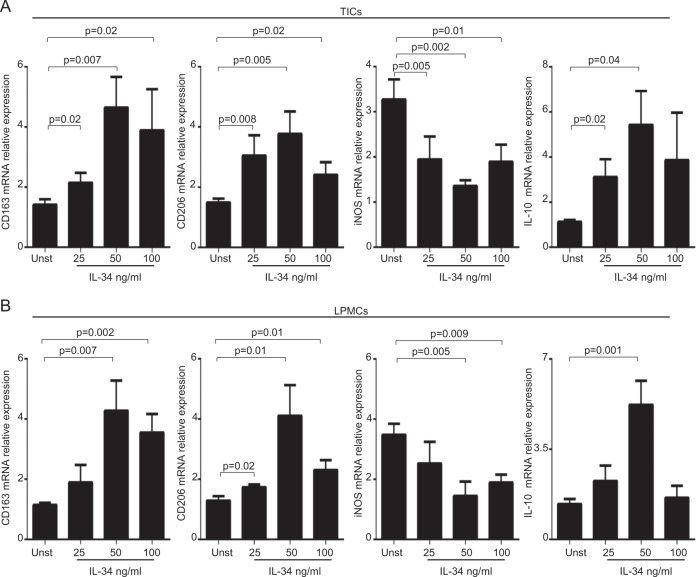
IL-34 enhances the RNA transcripts of markers of type 2-polarized macrophages. **A** Tumor-infiltrating cells (TICs) were either left unstimulated (Unst) or stimulated with increasing doses of human IL-34 (25–100 ng/ml) for 6 h. CD163, CD206, INOS, and IL-10 RNA transcripts were analyzed by real-time PCR, and levels were normalized to β-actin. Data are expressed as mean ± SEM of eight experiments. **B** LPMCs were isolated from non-tumoral samples of CRC patients and either left unstimulated (Unst) or stimulated with increasing doses of human IL-34 (25–100 ng/ml) for 6 h. CD163, CD206, INOS, and IL-10 RNA transcripts were analyzed by real-time PCR, and levels were normalized to β-actin. Data are expressed as mean ± SEM of five experiments.

**Fig. 6:**
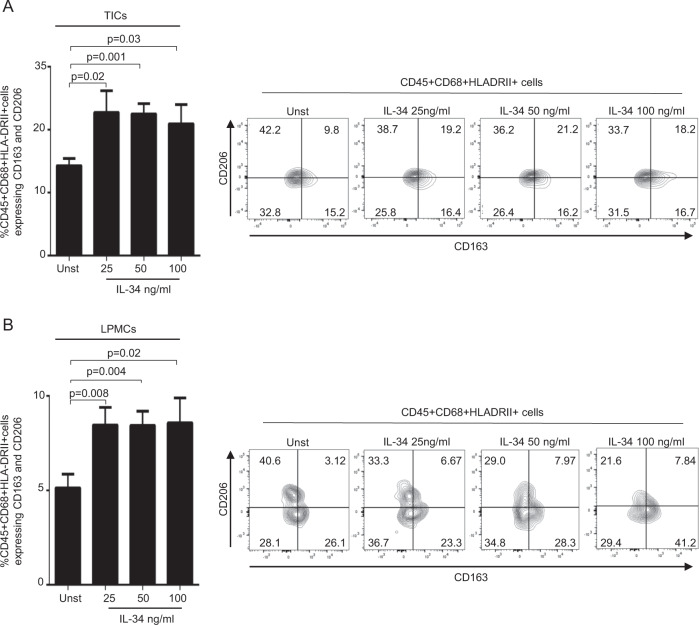
IL-34 enhances the expression of markers of type 2-polarized macrophages. **A**, TICs (**A**) LPMCs (**B**) were either left unstimulated (Unst) or stimulated with increasing doses of human IL-34 (25–100 ng/ml) for 24 h, and the percentages of live CD45 + , CD68 + ,HLA-DRII + cells expressing CD163 and CD206 were analyzed by flow cytometry. Data are expressed as mean ± SEM of seven experiments. Right panels: representative dotplots showing CD163 and CD206 expression in live CD45 + CD68 + HLA-DRII + TICs (**A**) or LPMCs (B) isolated from respectively tumoral and non-tumoral samples of one patient with colon rectal cancer (CRC) and treated as described above.

In subsequent experiments, we assessed whether IL-34 stimulates the synthesis of molecules, which are typically expressed by TAMs in CRC. To this end, we cultured both TICs and LPMCs with graded doses of recombinant human IL-34. At the end, cells were collected and assessed for the expression of CD163 and CD206, two markers of type 2-polarized macrophages^12^, INOS, a marker of type 1-polarized macrophages^12^ and IL-10, a cytokine involved in the control of type 2-polarized macrophages^13^. IL-34 significantly enhanced CD163, CD206, and IL-10 expression and decreased INOS expression in both TICs and LPMCs (Fig. 5A, B). Flow-cytometry analysis showed that, in unstimulated conditions, the percentage of CD68 + /HAL-DRII + cells expressing CD163 and CD206 was significantly greater in TICs (15 ± 1.1) than in LPMCs (5 ± 1; *P* < 0.05) (Fig. 6A, B). In line with the RNA results, stimulation of TICs and LPMCs with IL-34 increased significantly the fractions of CD163/CD206-expressing CD68 + HLA-DRII + cells (Fig. 6A, B).

### IL-34 enhances IL-6 expression in TAMs

**Fig. 7:**
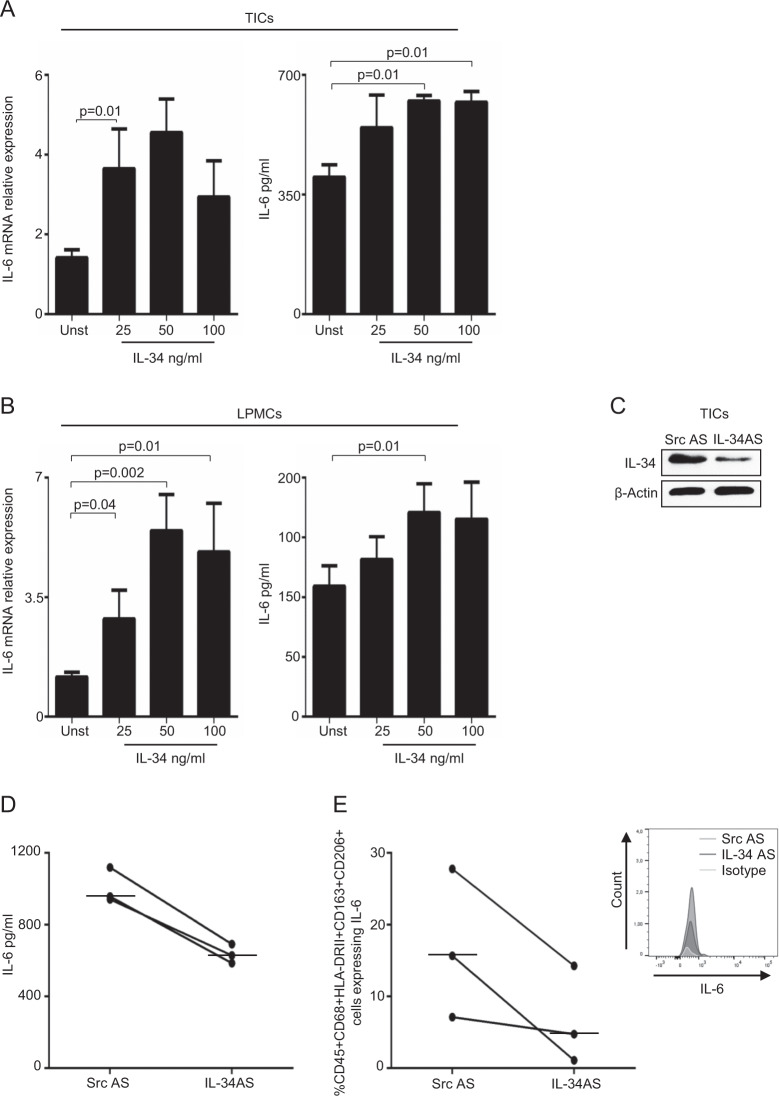
IL-34 increases IL-6 RNA and protein expression in both TICs and LPMCs. TICs (**A**) and LPMCs (B) were either left unstimulated (Unst) or stimulated with increasing doses of human IL-34 (25–100 ng/ml) for 6 h (left panel) or 48 h (right panel). Left panel: IL-6 RNA transcripts were analyzed by real-time PCR, levels were normalized to β-actin, and data are expressed as mean ± SEM of eight experiments. Right panel: IL-6 secretion was measured in the cell-free supernatants of TICs and LPMCs treated as above, and the data are expressed as mean ± SEM of eight experiments. **C** Intestinal TICs were isolated from tumoral samples of patients with CRC and transfected with a specific IL-34 antisense oligonucleotide (IL-34 AS) or with a scrambled antisense oligonucleotide (Src AS) (both used at 2 μg/ml) for 24 h. IL-34 and β-actin were analyzed by western blotting. One of three independent experiments is shown. **D** IL-6 secretion was measured in the supernatants of TICs isolated from tumoral samples of three patients with CRC and treated as described in **C** for 48 h. Horizontal bars indicate median values. **E** Intestinal TICs were isolated and treated as indicated in **C** and analyzed for the percentage of live CD45 + CD68 + HLA-DRII + CD163 + CD206 + cells expressing IL-6 (each point in the graph) by flow cytometry. Horizontal bars indicate the median values. Right panel: representative histogram showing IL-6 expression in live CD45 + CD68 + HLA-DRII + CD163 + CD206+ TICs isolated from tumoral samples of one patient with CRC, transfected with Src AS or IL-34 AS and analyzed by flow cytometry. Histogram with the respective isotype control antibody is also shown.

TAMs are supposed to participate in the progression and metastasis of CRC through the release of IL-6^14^. Therefore, we evaluated whether IL-34 enhanced macrophage-derived IL-6. Stimulation of both TICs and LPMCs with IL-34 increased IL-6 RNA transcripts (Fig. 7A, B left panel) and secretion (Fig. 7A, B right panel). Consistently, in three separate experiments, knockdown of IL-34 in TICs with a specific antisense oligonucleotide (AS) (Fig. 7C) decreased IL-6 secretion (Fig. 7D), as well as the percentage of CD163/CD206-expressing CD68 + HLA-DRII + cells producing IL-6 (Fig. 7E).

## Discussion

The molecular events that stimulate CRC cell growth are not fully understood. However, in recent years, a large body of evidence has been accumulated to show that the tumor microenvironment provides a variety of signals that sustain colon tumorigenesis. For instance, immune cells and stromal cells produce huge amounts of cytokines, which exert proliferative and survival effects on CRC cells^2^. In this context, we have recently shown that CRC cells synthesize IL-34, a factor that expands CRC cell growth^6^. Interestingly, both neoplastic cells and non-tumoral cells infiltrating CRC tissue express IL-34 receptors raising the possibility that IL-34 can mediate the cross-talk between cancer cells and other cells during colon carcinogenesis process^7^. This study was undertaken to examine whether IL-34 regulates the function of TAMs, as these cells are known to facilitate CRC progression^15^. Through flow-cytometry analysis of single cells isolated from both tumoral and non-tumoral specimens of CRC patients, we initially showed that both TAMs and normal mucosal CD68/HLA-DRII-positive macrophages expressed M-CSFR-1, a functional IL-34 receptor^16^. These findings confirm and expand on the data of previous studies showing that monocytes/macrophages are the main targets of IL-34^17^. Our data are also in line with studies documenting the expression of IL-34 receptors in TAMs in other settings^7^. Next, we showed that CD68/HLA-DRII-positive cells expressed IL-34 in both TICs and LPMCs preparations, even though the fraction of IL-34-positive cells was significantly higher in cancer samples. Interestingly, IL-34 was produced by CD68/HLA-DRII-positive cells either expressing or not M-CSFR-1, raising the possibility that this cytokine can regulate TAMs function by acting in a paracrine and/or autocrine manner. Indeed, stimulation of both TICs and LPMCs with IL-34 enhanced the expression of CD163 and CD206, typical markers of type 2 macrophages, a subset of cells that play critical roles in the promotion of tumor development, progression, metastasis, and therapeutic resistance^18^. These results well fit with the demonstration that expression of IL-34 is accompanied by increased infiltration of type 2-polarized TAMs in lung cancer^19,20^. Moreover, it has been demonstrated that IL-34 induces the differentiation of monocytes into IL-10^high^ IL-12^low^ immunoregulatory macrophages, which are similar to TAMs seen in ovarian cancer^17^. IL-34-treated macrophages switch non-Th17 committed memory CD4 + T cells into conventional CCR4 + CCR6 + CD161 + Th17 cells, a phenomenon which is typically seen in many cancer tissues^21^. In mammary cancer and other tumors (i.e., teratoma, hepatocellular carcinoma), the pro-tumoral effect of IL-34 has been linked to the ability of the cytokine to promote the function of type 2 macrophages^22–24^. Taken together, these findings suggest that, in CRC tissue, IL-34 contributes to maintain the function of type 2-polarized TAMs, with the downstream effect of sustaining colon carcinogenesis. The demonstration that IL-34 stimulates IL-6 induction in both TICs and LPMCs supports further the protumorigenic role of IL-34, as IL-6 can target directly cancer cells and activate signaling pathways that promote CRC cell growth and survival^25,26^.

In conclusion, this is the first to show a positive role of IL-34 in the control of TAMs in CRC, further supporting the hypothesis that IL-34 sustains colon tumorigenesis.

## Materials and methods

### Patients and samples

Paired tissue samples were taken from the tumoral area and the macroscopically and microscopically unaffected, adjacent colonic mucosa of 22 patients who underwent colon resection for sporadic CRC at the Tor Vergata University Hospital (Rome, Italy). All patients received neither radiotherapy nor chemotherapy prior to undergoing surgery.

Each patient who took part in the study gave written informed consent, and the study protocol was approved by the local Ethics Committee (Tor Vergata University Hospital, Rome. Protocol number:171/16).

### Cell isolation and culture

All the reagents were from Sigma-Aldrich (Milan, Italy) unless specified. TICs and LPMCs were isolated as previously described^27^ with the only exception that tissue digestion was performed with Liberase TM (200 μg/ml) and DNase I (200 μg/ml) (both Roche Diagnostics GmbH, Mannheim, Germany) instead of collagenase. Cells were resuspended in RPMI 1640 medium supplemented with 10% fetal bovine serum (FBS) and 1% of penicillin (P) (100 U/ml), streptomycin (S) (100 μg/ml), and gentamycin (G) (50 μg/ml) (all from Lonza Verviers, Belgium). Fresh TICs and LPMCs were phenotypically analyzed by flow cytometry or cultured in complete medium with PMA (40 ng ml/1), ionomycin (1 mg ml/1), and Brefeldin A (10 mg/ml) (Bioscience, San Diego, CA) for 4 h and then analyzed for IL-34 expression by flow cytometry.

To assess whether IL-34 regulates the function of type II-polarized macrophages, both TICs and LPMCs were seeded at a concentration of 5 × 10^5^ cells/ml into 48-well culture dishes with increasing doses of recombinant human IL-34 (25–100 ng/ml, Miltenyi Biotec, Bologna, Italy) for 6 or 24 h, and then analyzed by real-time PCR, ELISA, or flow cytometry.

In additional experiments, 5 × 10^5^ TICs or LPMCs were plated into each well of a 48-well plate, and then either left untreated or transfected with IL-34 AS or scrambled antisense oligonucleotide (Src AS) (both used a 2 µg/ml, Exiqon, Woburn, USA,) for 24–48 h using Opti-MEM medium and Lipofectamine 3000 reagent according to the manufacturer’s instructions (both from Life Technologies, Milan, Italy). The efficiency of the transfection was determined by western blotting after 24 h. To assess IL-6-producing cells, PMA (40 ng ml/1), ionomycin (1 mg ml/1), and Brefeldin A (10 mg/ml) were added 4 h before the end of the culture (24 h), and the cells were then analyzed by flow cytometry. Moreover, IL-6 was evaluated in cell-free supernatants by ELISA after 48-h culture.

### Real-time PCR

A constant amount of RNA (0,5 μg/sample) was retro-transcribed into complementary DNA (cDNA), and then 1 μl of cDNA/sample was amplified using the following conditions: denaturation 1 min at 95 °C; annealing 30 s, at 61 °C for β-actin and IL-6 and 62 °C for IL-10, followed by 30 s of extension at 72 °C. Primer sequences: IL-6: forward 5′-CCACTCACCTCTTCAGAACG-3’ and reverse 5′-GCCTCTTTGCTGCTTTCACAC-3’, IL-10 forward 5′-GGCACCCAGTCTGAGAACAG-3’ and reverse 5′- CTTGGCAACCCAGGTAACCC-3’; β-actin: forward, 5’-AAGATGACCCAGATCATGTTTGAGACC-3’ and reverse 5’-AGCCAGTCCAGACGCAGGAT-3’. mRNA expression was calculated relative to the housekeeping β-actin gene on the base of the ∆∆Ct algorithm. CD163, CD206, and INOS were evaluated using a commercial TaqMan probe (Applied Biosystems, Foster City, CA). RNA expression was calculated relative to the housekeeping β-actin gene on the base of the ∆∆Ct algorithm.

### Flow cytometry

Cells were stained with the following monoclonal anti-human antibodies: fluorescein isothiocyanate (FITC) anti-M-CSF1-R (R&D Systems, Minneapolis, MN, USA), allophycocyanin-H7 (APC-H7) anti-CD45, PerCP 5.5 anti-HLA-DRII, PerCP 5.5 anti-CD19, Pacific Blue anti-CD3, Alexa Fluor 647 (APC) anti-CD206, (all from Becton Dickinson, Milan, Italy), FITC anti-CD3 (Beckman and Coulter, Marseille, France), APC anti-CD68 (Biolegend, San Diego, California), phycoerythrin (PE) anti-CD68 (eBioscience, San Diego, CA), FITC anti-IL-6 (Miltenyi Biotec, Bologna Italia), PeCy7 anti-CD163 (Biolegend), PE anti-IL-34 (R&D Systems). In all experiments, appropriate isotype control IgG was used. All antibodies were used at 1:50 final dilution. For intracellular staining (CD68, IL-34, IL-6), cells were fixed and permeabilized using IC Fixation buffer and the permeabilization buffer (both from eBioscience) according to the manufacturer’s instruction. Cells were analyzed by flow cytometry (FACSVerse, Becton Dickinson), and analysis was performed by acquiring 10,000 events in the gate of CD45 + live cells according to LIVE/DEAD staining (Life Technologies, Milan, Italy). Flow-cytometry data were analyzed by FlowJo ver. 10.7 (Becton Dickinson).

### Immunohistochemistry

Immunohistochemistry was performed on formalin-fixed, paraffin-embedded colon sections of five CRC patients. The sections were deparaffinized and dehydrated through xylene and ethanol, and the antigen retrieval was performed in Tris EDTA citrate buffer (pH 7.8) in a thermostatic bath at 98 °C for 30 min. Immunohistochemical staining was performed using mouse monoclonal antibody directed against human IL-34 (final dilution 1:50000, Abcam, Cambridge, UK), and a mouse monoclonal antibody directed against human CD68 (final concentration 1:100, Dako, Milan, Italy) incubated at room temperature (RT) for 1 h followed by a biotin-free HRP-polymer detection technology with 3,3’diaminobenzidine (DAB) as a chromogen (MACH 4 Universal HRP-Polymer Kit, Biocare Medical). The sections were counterstained with hematoxylin, dehydrated, and mounted. Isotype control IgG-stained sections were prepared under identical immunohistochemical conditions as described above, replacing the primary antibody with a purified mouse normal IgG control antibody (R&D Systems). The IL-34 and CD68-positive cells were counted in at least six fields per section using IAS 2000 System (Delta Sistemi, Rome, Italy) in serial sections of the same CRC surgical samples and expressed as the number of cells for high-power field (hpf).

### Immunofluorescence

Immunofluorescence was performed on frozen sections of mucosal samples taken from the colon sections of three CRC patients. Samples were embedded in a cryostat mounting medium (Neg–50 Frozen Section Medium, Thermo Scientific), snap-frozen, and stored at −80 °C. Sections (6-µm thick) were mounted onto superfrost glass slides and fixed in 4% paraformaldehyde (PFA) for 10 min at 4 °C. Slides were washed three times with PBS, treated with 0.1% Triton X-100 for 20 min at RT. The blocking procedure was performed with 10% normal goat serum in PBS solution for 1 h at room temperature. Slides were then incubated overnight at 4 °C with mouse anti-human IL-34 (final dilution 1:100, Abcam, Cambridge, UK), rabbit anti-human CD68 (final dilution 1:100, BioSpring Germany, Frankfurt). After washing three times with PBS, slides were incubated for 1 h at room temperature with specific secondary antibodies coupled with Alexa Fluor Dyes (final dilution 1:2000 final dilution; Invitrogen, Milan, Italy). Coverslips were mounted on glass slides using ProLong Gold antifade reagent with DAPI (Invitrogen) to counterstain the DNA. Isotype control IgG-stained sections were prepared under identical immunofluorescence conditions as described above, replacing the primary antibody with a normal IgG control antibody (Abcam). Samples were analyzed with a Leica DMI 4000 B fluorescence microscope (Leica, Wetzlar, Germany).

### Enzyme-linked immunosorbent assay

Human IL-6 was measured using a sensitive commercial ELISA kit (R&D Systems) according to the manufacturer’s instructions.

### Statistical analysis

Differences between the two groups were compared using the Student’s *t* test or Mann–Whitney *U* test. The significance of correlation was determined using Pearson’s test. All the analyses were performed using Graph-Pad 6 software.
